# Climate and fragment area jointly affect the annual dynamics of seedlings in different functional groups in the Thousand Island Lake

**DOI:** 10.3389/fpls.2023.1200520

**Published:** 2023-06-14

**Authors:** Yuping Zhong, Yuchen Zhong, Yuchu Xie, Yanping Lei, Boliang Wei, Jinliang Liu, Mingjian Yu

**Affiliations:** ^1^ College of Life Sciences, Zhejiang University, Hangzhou, China; ^2^ Hangzhou Xuejun High School, Hangzhou, China; ^3^ Wuyanling National Nature Reserve Administration of Zhejiang, Wenzhou, China; ^4^ College of Life and Environmental Science, Wenzhou University, Wenzhou, China

**Keywords:** fragmentation, species coexistence, habitat filtering, regeneration niche difference, seed-to-seedling transition, seedling recruitment, seedling survival

## Abstract

Habitat fragmentation and climate change are the two main threats to global biodiversity. Understanding their combined impact on plant community regeneration is vital for predicting future forest structure and conserving biodiversity. This study monitored the seed production, seedling recruitment and mortality of woody plants in the Thousand Island Lake, a highly fragmented anthropogenic archipelago, for 5 years. We analyzed the seed-seedling transition, seedling recruitment and mortality of different functional groups in the fragmented forests and conducted correlation analyses involving climatic factors, island area, and plant community abundance. Our results showed that: 1) shade-tolerant and evergreen species had higher seed-seedling transition, seedling recruitment and survival rate than shade-intolerant and deciduous species in time and space, and these advantages increased with the island area. 2) Seedlings in different functional groups responded differently to island area, temperature and precipitation. 3) Increasing active accumulated temperature (the sum of the mean daily temperature above 0 °C) significantly increased seedling recruitment and survival, and warming climate favored the regeneration of evergreen species. 4) The seedling mortality rate of all plant functional groups increased with the increase of island area, but the increasing strength weakened significantly with the increase of the annual maximum temperature. These results suggested that the dynamics of woody plant seedlings varied among functional groups, and can be regulated separately and jointly by fragmentation and climate.

## 1 Introduction

Species diversity varies across time and space, and studying species diversity and its driving factors is a primary goal of ecology (Rosenzweig and Rosenzweig, 1995). Globally, at least half of natural habitats have been lost (Diversity, S.o.t.C.o.B, 2020), and the remaining habitat fragments are usually small (Liu et al., 2019a), suffering from edge effect. Meanwhile, the climate is changing into a warmer and drier condition, which is potentially affecting forest regeneration and seedling dynamics (Bond-Lamberty et al., 2014; Ibanez et al., 2017; Badano and Sánchez-Montes de Oca, 2022). Edge effect follow by fragmentation can cause increasing temperature extremes and a drier microclimate inside small fragments (Saunders et al., 1991; Debinski and Holt, 2000), enhancing the influence of climate change on seedling dynamics. However, there is still limited research considering the potential interactive effects of climate and fragmentation on plant community dynamics (Opdam and Wascher, 2004; Mantyka-Pringle et al., 2011).

Seedlings face various abiotic and biotic factors that significantly reduce their recruitment and survival rates (Harper, 1977; Leck et al., 1989; Moles and Westoby, 2004). The seedling stage is considered a critical bottleneck in population growth rate due to high mortality rates (Grubb, 1977; Harper, 1977). The success of seedling recruitment, survival and establishment is essential to plant population dynamics and ultimately affects the composition and sustainability of the community (Grubb, 1977; Swaine, 1996; Baeten et al., 2009). Therefore, understanding seedling dynamics is crucial in predicting short-term changes of plant communities in a fragmented landscape (Bykova et al., 2012; Rother et al., 2013; Kroiss and Hillerslambers, 2015).

Fragmented habitats are usually relatively small in size, creating many edges that alter the microclimate in various ways that impairs the regeneration of certain tree species (Grubb, 1977; Swaine, 1996; Tabarelli et al., 2005). Small fragments tend to suffer from more severe edge effects, resulting in less humidity, more light resources, and greater temperature changes, which can penetrate up to 60 m inside the fragments (Kapos, 1989). Studies found that fragmentation has changed the regeneration dynamics of vegetation community (Wilcox and Murphy, 1985; Sarmiento, 1997; Turner et al., 1998; Laurance et al., 2000), leading to the transformation of the seedling community to species-poor composition, threatening forest biodiversity (Benitez-Malvido and Martinez-Ramos, 2003). Furthermore, the defaunation of large animals caused by habitat loss can potentially alter important biotic interactions, such as seed predation and seedling herbivory (Harrison et al., 2013; Dirzo et al., 2014).

Climate can also alter the emergence and establishment of tree seedlings. For instance, species with shallow-rooted seedlings may experience limited seed germination and increased seedling mortality during drought (Clark et al., 2016). Higher temperatures and lower precipitation levels can intensify seed predation (McKone et al., 1998; Hillyer and Silman, 2010), altering the water and carbon balance of tree seedlings, resulting in a reduced seedling emergence (Joet et al., 2013) and impair their establishment (Perez-Ramos et al., 2013; Perez-Ruiz et al., 2018). Studies have shown that variations in climatic conditions lead to interannual fluctuations in the quality of microhabitat of seedling recruitment (Greenlee and Callaway, 1996; Tielborger and Kadmon, 2000; Ibanez and Schupp, 2001).

With the intensification of climate change and fragmentation, the interaction of these two main disturbances on altering the regeneration process of communities is attracting attention. Their combined impacts may have hidden effects that are not evident when studied in isolation. It was found that the fragment area affects the size and composition of the soil seed bank (Sousa et al., 2017), and successful seed dispersal, seedling germination and survival are limited by the fragmentation of suitable habitats (Pitelka and the Plant Migration Workshop Group, 1997; Renton et al., 2013) and are directly affected by climate change. Due to the different seedling dynamics of different tree species (e.g., functional groups) and their response to climate change, habitat fragmentation can interact with climate on seedling dynamics by affecting soil seed banks (and microenvironments). However, limited by field research platforms and systems and the accumulation of long-term monitoring data, few people pay attention to the combined effects of habitat fragmentation and climate change (Opdam and Wascher, 2004).

The maintenance of species diversity is the central question of community ecology, which concerns the coexistence of large numbers of species (Hutchinson, 1961; Hart et al., 2017). Regeneration niche differentiation in early stages of seedling germination and establishment, and negative density dependent (NDD) are two important mechanisms to explain species coexistence and biodiversity maintenance (Janzen, 1970; Connell, 1971; Grubb, 1977; Denslow, 1980; Bagchi et al., 2014). Along the gradient of environmental resources, changes in seedling survival rates and growth rates of different functional groups will lead to their regeneration niche differentiation (Grubb, 1977) and promote species coexistence at the landscape level (Macarthur and Levins, 1967; Chesson, 2000). Local scale NDD can result in community compensatory trend (CCT), which describes the negative relationship between species abundance and population growth rate at the community level. CCT prevents common species from overtaking the ecological niche of rare species, thereby promoting their coexistence (Connell et al., 1984).

The growth and regeneration dynamics of different plant functional groups with different resource requirements are affected by the level of light and other resources, which differ between internal and marginal forests. Base on their light tolerance, plants can be classified as shade-tolerant (ST) and shade-intolerant (SI) species, and as evergreen (EG) and deciduous (DC) species based on their leaf habit. Usually, the light intensity in large patches and internal forests is very low due to the closed canopy, which is very similar to an intact habitat. As fragment area decreases, the light intensity gradually increases, limiting the regeneration of ST species. DC and EG species commonly coexist in subtropical EG broad-leaved forests, although a longer growing season should favor species with long leaf lifespans (Givnish, 2002). Some studies have proposed the heterogeneity of topography (Tang and Ohsawa, 2002; Fang et al., 2017) and light intensity may cause niche differentiation of EG and DC species and promote their coexistence (Jin et al., 2018). Compared with DC species, seedlings of EG species are usually more shade-tolerant (Wang et al., 2007; Baldocchi et al., 2010; Kitajima et al., 2013; Jin et al., 2018), and previous research have found that small fragments and forest edges tend to have more SI and DC species (Slik et al., 2008; Tabarelli et al., 2010; Liu et al., 2019c). The species and functional composition of forest communities change with the fragmentation and forest successional stage (Laurance et al., 2006; Slik et al., 2008; Kooyman et al., 2013; Martin et al., 2014). Since it may be the most relative demographic information to predict future forest composition and succession processes, however, there is still very little research on the regeneration dynamics of secondary forests over time and space in fragmented landscapes.

To better understand the mechanism of plant diversity under climate and habitat fragmentation, it is necessary to explore the differences in regeneration dynamics and their driving factors between different functional groups in fragmented forests. Our research focused on secondary forests in the Thousand Island Lake. In this study, we aim to explore the seedling dynamic differences between different functional groups, and analyze how climate change and fragmentation influence these patterns. Specifically, we answer the following questions: 1) Do seedlings of ST and EG species exhibit higher demographic advantages on larger islands compared with SI and DC species? 2) Does it have significant effects on the seedling dynamics of different functional groups? 3) Is there an interaction between climate change and fragmentation on seedling dynamics?

## 2 Methods

### 2.1 Study site

The study was conducted on islands in the Thousand Island Lake of Zhejiang Province, China (29°22′– 29°50′N and 118°34′– 119°15′E). The lake is a hydroelectric reservoir formed by the construction of the Xin’an River Dam in 1959. After that, the valley (around 573 km^2^) was flooded, forming more than 1,000 land- bridge islands larger than 0.25 ha (Yu et al., 2012; Liu et al., 2018; Hu et al., 2021). Forests were clear- cut before the construction. After 60 years of succession, most of these islands are now covered by secondary forests dominated by Masson pine (*Pinus massoniana*) and mixed broad- leaved species (Liu et al., 2019b). The climatic conditions of the study site are typical of the central subtropical climate zone, with an average annual temperature of 17.0°C (the daily temperature ranges from -7.6 °C in January to 41.8 °C in July) and an average annual precipitation of 1,430 mm (Hu et al., 2011).

### 2.2 Tree, seed and seedling census

From 2009 to 2010, we established a long-term plant community monitoring site of 12.7 ha on 29 sample islands spanning available variation in area (**Supplementary Table S1**). Quadrats were set all over the islands with area smaller than 1 ha. One to several transects with a wide of 20-40 m were set on islands with area larger than 1 ha, and the total area of transects were designed based on its island area. We defined islands with area smaller than 1 ha as ‘small island’ (s), area larger than 1 ha but smaller than 10 ha as ‘medium island’ (m), and area larger than 10 ha as ‘large island’ (l). Woody plants with DBH above 1 cm were investigated and recorded, and the first census was completed in 2014-2015. The second census was completed in 2019. Community abundance refers to the number of individual of species on each island, which was calculated based on this census.

Seedling plots were set base on random sampling by classification. Within each sample island, 1 m×1 m seedling plots were randomly set from the edge to the interior on island, and the interval between plots was at least 5 m. The seedling plots evenly covered different edge gradients from the edge to the center of the transect, to fully understand the composition and dynamics of the seedling communities on the island. A total of 499 seedling plots have been set up (**Supplementary Table S1**). In April 2017, all woody seedlings in the seedling plots were tagged with a unique number, identified to species and then mapped. The census was conducted in April and September each year from 2018-2022. During each census, we counted and identified all the seedlings in the plot, measured their height and counted their leaf number. newly recruited seedlings were tagged, and missing seedlings were recorded as ‘dead’. Here, ‘seedlings’ refer to plants with DBH< 1 cm and height< 20 cm (Comita et al., 2007).

A total of 240 seed-traps of 0.5 m^2^ were set up, and each trap was located 2 meters away from the seedling plot. Since the seedling recruitment rate is calculated based on the data of the seedling of the current year and the seed rain of the previous year (Peña-Domene et al., 2016), fruits and seeds in the seed-traps were collected monthly from January 2017 to December 2021. After drying, they were identified to species, counted and weighed. Numbers of seedling plots and seed-traps set on each island are listed in **Supplementary Table** (**Supplementary Table S1**).

### 2.3 Climate data

The monthly climate data were obtained from the nearest Chun ‘an Meteorological Station (located approximately 17 km north-east of the study site). We calculated 6 climate indexes, including the annual maximum temperature (*T_max_*), the annual minimum temperature, the annual mean temperature, the annual active accumulated temperature (The sum of temperatures ≥0°C, *T_active_*), the annual precipitation (*P*_total_) and the sunshine duration.

### 2.4 Calculations for dynamic index

The shade tolerance and leaf habit of all species were derived from the results of previous studies conducted in the TIL (Tian et al., 2016; Hu et al., 2011), and indexed through the Flora of China (FOC, http://www.iplant.cn/foc) website. In total, we conducted 50 plant species, Overall, there were 22 EG species, 28 DC species, 22 SI species and 28 ST species (**Supplementary Table S2**). Since the evolutionary history of gymnosperms is much longer than that of angiosperms, the response of certain functional traits in angiosperms cannot be extrapolated to gymnosperms (Aiba et al., 2016). Including both gymnosperms and angiosperms in our analysis may obscure the overall pattern. Therefore, 3 gymnosperms species were excluded from the main analysis. An analysis of gymnosperms species that were not excluded was also carried out and showed in the supplement (**Supplementary Figures S1–S4**).

The ratio of seeds to seedlings (seed effectiveness; Φ_i_) is the number of seeds of species i needed for 1 recruit (Peña-Domene et al., 2016). Here, we defined the seed-seedling transition rate as the number of seedlings of the species i successfully germinated from 1 seed:


$$T_{i}=\frac{r_{i}}{A\times S_{i}}$$

where $S_{i}$ is the total number of seeds of species i that fall in the trap of a habitat, $r_{i}$ is the total number of recruits of species i in a habitat, and A is the seedling plot area (m^2^) sampled in a habitat divided by the area sampled by seed traps in a habitat.

The seedling recruitment rate is the number of seedlings that appeared on the ground for the first time during the census divided by the total number of seedlings that appeared during the last census:


$$R_{i}=\frac{r_{i}}{d_{i0}}$$

The seedling mortality rate:


$$M_{i}=\frac{d_{i}-d_{i0}}{d_{i0}}$$

where $M_{i}$ is the seedling mortality rate of species i in a habitat, $d_{i}$ is the number of seedlings of species i in a habitat (excluding new recruits), and $d_{i0}$ is the total number of seedlings of species i in a habitat during the last census.

### 2.5 Statistical analysis

The Wilcoxon test was used to test the differences in seedling dynamics on different islands, using ‘wilcoxon_test’ function in package ‘rstatix’.

The correlation between 6 year-level climate variables pairwise was calculated using the function ‘cor’, and then the ‘findCorrelation’ function in the package ‘caret’ was used to remove variables with a mean absolute correlation value of more than 4. Three climate variables were selected from six variables, which are *T_max_*, *P*_total_ and *T_active_*. The island area refers to the projected area of the island when the water reaches its highest level (105 meters above sea level), which was calculated using ArcGIS software. Log transformation of the island area was performed because the data vary a lot on the relative scale. The fixed effects of the models included three climatic factors (*T_max_*, *P*_total_ and *T_active_*), community abundance (*Abun*) and the island area (*Area*), which were scaled and centered using the function ‘scale’ before regression. Then, we used the General Liner-Mix Model (GLMM) to assess the influence of the 5 predictors on the seedling dynamic index (transition, recruitment or mortality rate) by generating a Template Model Builder (TMB) (package ‘glmmTMB’). To account for interspecies differences, the species was considered a random effect in the model. The model was summarized as follows:


$$\begin{array}{c} D_{ijk}=\beta_{0}+\beta_{1}\times Abun_{i}+\beta_{2}\times Area_{j}+\beta_{3}\times T_{max\_k\;}+\\ \beta_{4}\times P_{total\_k}+\beta_{5}\times T_{active\_k}+\varphi_{i} \end{array}$$

where $D_{ijk}$ is the seedling dynamic (transition, recruitment or mortality rate) of species i in island j in year k. The parameter $\beta_{0}$ represents the intercept, $\beta_{1}$ , $\beta_{2}$ , $\beta_{3}$ , $\beta_{4}$ and $\beta_{5}$ represent the effect of 5 factors, respectively; $\varphi_{i}\;$ represents the random effect of the species.

To answer whether climate change has modified the effects of fragmentation on seedling dynamics, we used GLMM to quantify the effect of island area on seedling dynamics, and species and census year were considered as random effects. We defined the correlation slope between island area and seedling dynamics as ‘*z*’ index. The liner regression model (LM) was used to analyze the effects of each climatic factor on the *z*-index, using the census year as the time offset. Since the seedling dynamics are calculated based on the previous year’s census data, the inclusion of time offsets can capture the dynamics and dependencies that exist in the time series data. By incorporating a time offset into the model, we can improve the model’s ability to make accurate estimates.

All the analyze above were generated in R4.2.1.

## 3 Results

### 3.1 Temporal and spatial dynamic of seedling

**Figure 1:**
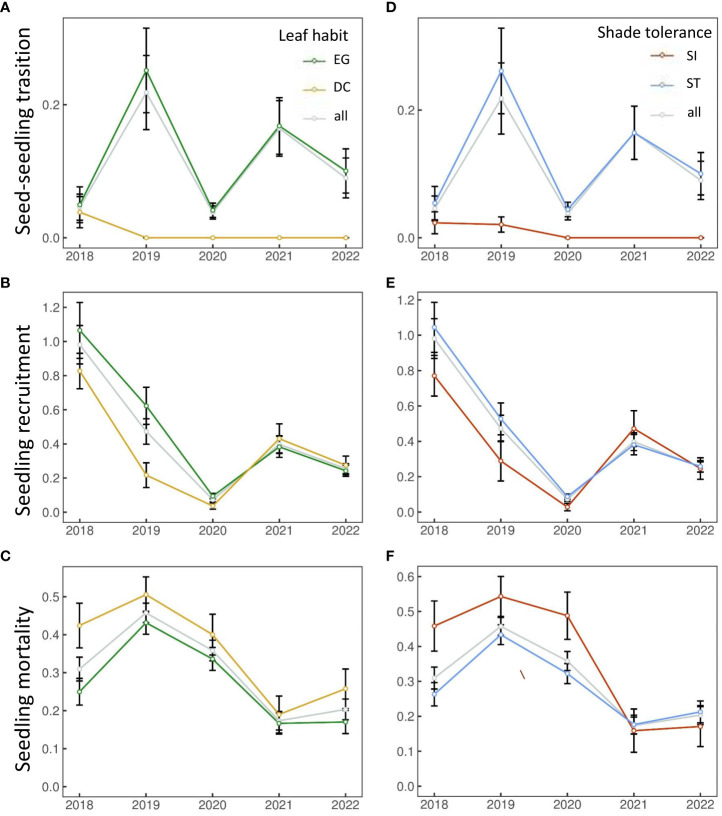
Seedling dynamics [seed-seedling transition **(A, D)**, seedling recruitment **(B, E)** and seedling mortality **(C, F)**] from 2018 to 2022.

The number of seedling individuals in each functional group showed interannual fluctuations within 5 years. The number of seedling individuals has decreased significantly in 2021-2022 compared with the previous three years (**Table 1**). Seedling dynamics also showed interannual fluctuations, and the transition rate and mortality rate did not increase or decrease significantly. Seedling recruitment exhibited a decreasing trend (**Figures 1B, E**). Overall, EG and ST species showed higher transitions and recruitment rates (**Figures 1A, B, D, E**), and the mortality rate was lower than that of DC and SI species (**Figures 1C, F**), respectively. The recruitment of EG and DC species has been converging since 2020 (**Figure 1B**). Similar trends were shown in ST and SI species (**Figure 1E**).

**Table 1:** Total number of seedling individuals of different functional groups on all islands in 5 years.

	2018	2019	2020	2021	2022
**Deciduous**	798	1089	831	404	387
**Evergreen**	465	594	459	269	246
**Tolerant**	739	955	766	402	349
**Intolerant**	524	728	524	271	284
**Total**	1263	1683	1290	673	633

**Figure 2:**
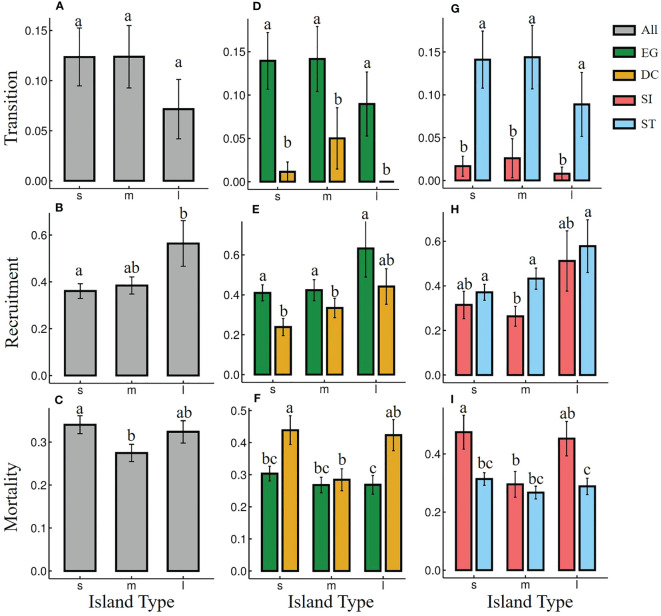
Seedling dynamics [seed-seedling transition **(A, D, G)**, seedling recruitment **(B, E, H)** and seedling mortality **(C, F, I)**] of different functional groups on islands with different area from 2018 to 2022. “s”, “m”, and “l” represent small, medium and large islands, respectively. Seedling dynamic of all species (grey), evergreen (EG, green) and deciduous (DC, yellow) species, Shade-intolerance (SI, red) and shade-tolerance (ST, blue) species were shown. Different letters between functional groups or island types indicated significant differences.

There was no significant difference in most seedling dynamic index among most island types (**Figures 2A–C**), except for the seedling mortality rates of DC and SI species between small and medium islands (**Figures 2F, I**). EG species showed significantly higher transitions and recruitment rates, and lower mortality rate than that of DC species on almost all types of islands (**Figures 2D–F**). ST species showed significantly higher transitions and lower mortality rates than SI species on almost all types of islands (**Figures 2G–I**). The seedling recruitment of ST species was significantly higher than that of SI only on medium islands (**Figure 2H**).

### 3.2 Abiotic and biotic effects on seedling dynamics

**Figure 3:**
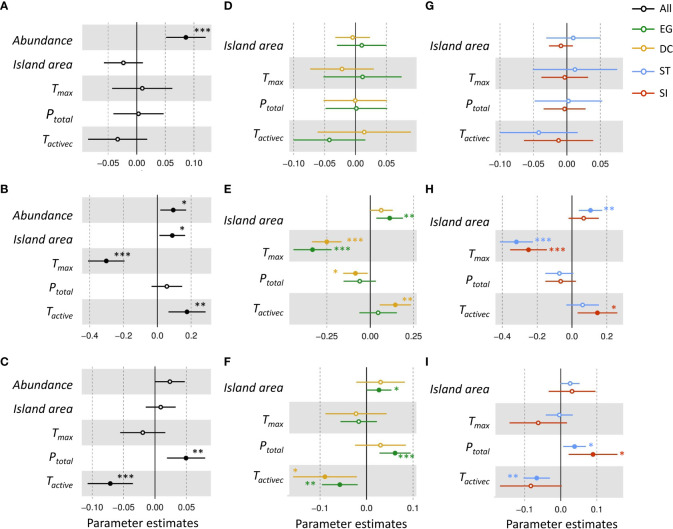
The relative influence of potential factors [the community abundance (*Abundance*), island area, annual maximum temperature (*T_max_*), annual precipitation (*P_total_*) and active accumulated temperature (*T_active_*)] on the transition **(A, D, G)**, recruitment **(B, E, H)** and mortality **(C, F, I)** rate of seedlings of evergreen(EG, green), deciduous(DC, yellow), shade-intolerat(SI, red), shade-tolerant(ST, blue), and all (black) species. Solid points with asterisk marks indicate a significant effect (significant codes for *p*-value: *p*= 0~ 0.001 “***”, *p*= 0.001~ 0.01 “**”, *p*= 0.01~ 0.05 “*”). If the coefficient value of the explanatory variable is<0, it means a significant negative correlation. The parameter estimate<0 indicated a significant negative effect. The parameter estimate intersects with 0 indicated an insignificant effect. The parameter estimate >0 indicates a significant positive effect.

Tree abundance had a positive effect on seed-seedling transition rate and seedling recruitment (**Figures 3A–C**). Island area positively affected seedling recruitment of EG and SI species, and increased the mortality rate of EG seedlings (**Figures 3D–I**).

The increase in *T_active_* significantly increased the seedling recruitment of DC seedlings and reduced seedling mortality to a greater extent than that of EG species (**Figures 3E, F**). For all functional groups, the increase in *T_max_* significantly decreased seedling recruitment, but had no effect on seedling mortality. The increase in *P*_total_ significantly decreased seedling recruitment of DC species and increased seedling mortality in most functional groups (**Figures 3E, F, H, I**).

### 3.3 Interaction effects of climate and island area on seedling dynamics

**Figure 4:**
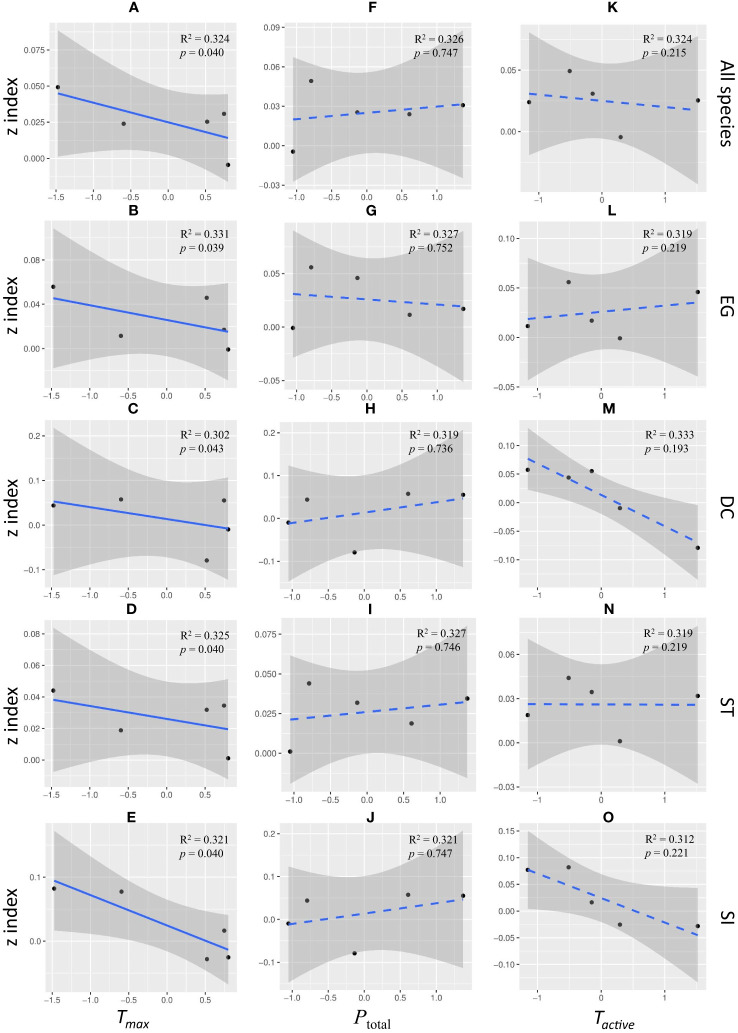
Linear regression correlation between *z*-value and annual maximum temperature (*T_max_*: **(A–E)**, annual precipitation (*P_total_*: **(F–J)**, and the annual active accumulated temperature (*T_active_*: **(K–O)** for all species **(A, F, K)**, evergreen species **(B, G, L)**, deciduous species **(C, H, M)**, shade-tolerant species **(D, I, N)**, and shade-intolerant species **(E, J, O)**. The five points in the panels represent the *z*-index values calculated based on year-level climatic factors during 5 years. To be noted, the *p*-value in this figure was calculated based on the results of LM with time offset.

The increase rate in seedling mortality with island area weakened significantly with the increase of *T_max_* in all functional groups. There was a negative correlation between the overall mortality *z* index and *T_max_* (**Figure 4A**, slope = -1.431, *R^2^* =0.324, *p*< 0.05). Same pattern were showed in EG species (**Figure 4B**, slope = -1.430, *R^2^* =0.331, *p*< 0.05), DC species (**Figure 4C**, slope = -1.443, *R^2^* =0.302, *p*< 0.05), ST species (**Figure 4D**, slope = -1.425, *R^2^* =0.325, *p*< 0.05) and SI species (**Figure 4E**, slope = -1.442, *R^2^* =0.321, *p*< 0.05). There was no significant correlation between the mortality *z* index and *T_active_* or precipitation (**Figures 4F–O**, *p*>0.05).

## 4 Discussion

Our study provides insights into seedling dynamics and the influence of environmental variables on these dynamics in fragmented forests. The results showed that the interaction of climatic factors and island area affected the dynamics of seedlings in different functional groups, which can explain the composition patterns of plant communities. The joint effects of climate change and fragmentation can continue to shape the community structure and local biodiversity of the area.

In this research, seedling dynamics showed interannual fluctuations. EG and DC seedlings, ST and SI seedlings showed very similar mortality and recruitment trend in space and time, but EG and ST species showed higher seed-seedling transition, recruitment and survival rate over DC and SI species in most island types and most years, suggesting that they have a competitive advantage in the early life stage. This is consistent with previous research, showing that EG seedlings have a regenerative advantage over DC seedlings (Jin et al., 2018), the same as ST over SI (Tian et al., 2016). With the progress of community succession, EG species and ST species may gradually develop into dominant species in the area.

Island area significantly affected seedling mortality and recruitment. The island area had no effect on the seed- seedling transition, but it had a positive impact on the seedling recruitment and mortality of EG species, and also on the seedling recruitment of ST species. Small forest fragments are suffering from more severe edge effect than large fragments, with greater temperature and low humidity (Laurance and Yensen, 1991; Malcolm, 1994; Fahrig, 2003; Ewers and Didham, 2006; Porensky, 2011; Laurance et al., 2018). These conditions compromise seed-seedling transition and seedling establishment of ST species (Benitez-Malvido, 1998; Bruna, 1999; González-Di Pierro et al., 2011), causing small fragments to have fewer ST species (Slik et al., 2008). Combined with the spatiotemporal seedling dynamics of EG and DC species, it is predicted that the increase in habitat area may favor the regeneration of EG and ST species, which may g radually lead to their dominant position in the community. Thus, habitat loss followed by fragmentation may suppress the regeneration of EG and ST species, limiting their potential to become dominant species.

Temperature and precipitation significantly affect seedling recruitment and mortality, but the response varied across different functional groups. We found that higher *T_active_*, lower *T_max_* and precipitation significantly increased the seedling recruitment and survival. This result indicates that extremely high temperatures can reduce the seedling recruitment, but warming with smaller seasonal temperature changes can potentially increase seedling recruitment and survival rate as increase of the active accumulated temperature may prolong the growth time of seedlings. This is partially in line with the finding saying that increased warming and drought may potentially decrease seedling germination, emergence and establishment (Ibanez et al., 2017; Badano and Sánchez-Montes de Oca, 2022). Studies have found that warmer and drier conditions can reduce the survival of tree seedlings by altering the water and carbon balance (Perez-Ruiz et al., 2018; Badano and Sánchez-Montes de Oca, 2022). But some researches have also found that the seedling mortality rate of some species has declined with warming (Chidumayo, 2008). Hence, the seedling dynamics response to climate warming varies from species and region to region. Higher levels of precipitation also increased seedling mortality. The increase in mortality with the increase of precipitation may be caused by its promotion of pathogen and herbivory damage to seedlings, as found in previous research (Milici et al., 2020; Ebeling et al., 2021).

The results also showed that seedlings in different functional groups responded differently to climate factors. As found in previous studies, distributions of EG and DC tree species are determined by precipitation and temperature (Barbosa et al., 2014), and elevated temperatures enhanced the growth of DC trees more than that of EG trees (Way and Oren, 2010; Barbosa et al., 2014). Our results also showed similar results. We found that increasing *T_active_* promoted the seedling recruitment of DC species and reduced their mortality to a greater extent compared with EG species.

However, we did not detect CCT in community level in the study. Our results showed that species abundance significantly increased the overall seed-seedling transition and seedling recruitment. This suggested that species with higher abundance in the community have a better advantage in germination and recruitment success, which does not support CCT. However, some other studies have also found significant positive relationships between seedling survival and adult conspecific density (Inman-Narahari et al., 2016). Studies found that in the case of warming climate, the negative density effect in seedling survival becomes positive (Bachelot et al., 2020). The stress gradient hypothesis (SGH) (Bertness and Callaway, 1994) predicts that interspecific species interactions should shift from negative to positive with environmental stress, and similar shifts have been found in intraspecific interactions, which can explain the regenerative advantages of abundant species in this region with fragmentation as an important stressor.

Most importantly, our study suggests that the increase rate in mortality with island area weakens significantly as the annual maximum temperature increases. Fragmentation can exacerbate the effects of climate change on plant and animal communities through various mechanisms (Davies et al., 2004; Fahrig, 2003). If the potential joint impacts are greater than the individually estimated impacts, separate research on climate change and fragmentation may be misleading (De Chazal and Rounsevell, 2009). However, the joint effects of these two driving mechanisms are not well understood. In our results, although there was no direct significant effect of *T_max_* on seedling mortality, our study did find a negative correlation between mortality *z*-index and *T_max_*, indicating that higher temperatures could either increase seedling mortality in smaller islands or mitigate it in larger islands. In large fragments, a closed canopy can increase seedling mortality by reducing light penetration (Matlack, 1994; Camargo and Kapos, 1995) as well as increasing litterfall and debris that may damage seedlings (Coley et al., 1985; Sizer, 1992; Benitez-Malvido, 1998). Small islands generally have a more open canopy and a higher proportion of edge area. Additionally, smaller islands are usually drier and warmer, making them more susceptible to external climate change (Laurance, 2004). Consequently, the increase in *T_max_* may have a greater negative impact on seedling survival on relatively small islands compared to large islands. Considering the projected increase in high maximum temperatures in the future, we anticipate that the negative effect of high maximum temperatures on the seedling survival on small fragments may offset their survival advantage over large fragments. This is consistent with a previous meta-analysis, indicating that the effects of habitat loss and fragmentation are most pronounced in areas with high maximum temperatures (Mantyka-Pringle et al., 2011).

However, this study still has some limitations. Our study did not explore how the interaction of climatic factors and island area influences the local environment or identify direct factors affecting seedling regeneration. Climate parameters were based on regional scales, while seedling demographic dynamics were based on island scales or even plot scales. This will probably mask a lot of real variations and real environmental filtering effects. Future research should give priority to monitoring the microenvironment (light, water, temperature) of seedlings to further understand the relationship between climate change, island area, island microhabitat and seedling dynamics.

## 5 Conclusions

In conclusion, this study provides insights into seedling dynamics and the effects of environmental variables on these dynamics in fragmented forests. The results suggest that shade tolerance of species, island area, and climate variables, such as temperature and precipitation, significantly influence the seedling dynamics of woody plants. More importantly, we found that fragmentation and temperature jointly affect seedling mortality. These findings can provide important implications for understanding the basic mechanisms of plant community dynamics, and can give informations for management strategies aimed at promoting the regeneration of plant communities in fragmented habitats.

## Data availability statement

The original contributions presented in the study are included in the article/**Supplementary Material**, further inquiries can be directed to the corresponding author/s.

## Author contributions

YPZ, YCZ, YL collected the data. YPZ conducted the formal analysis, wrote and prepared the original draft.; YPZ, JL, BW, YX, YL, and MY reviewed and edited. Supervision and funding acquisition, MY. All authors have read and agreed to the published version of the manuscript.

## References

[B1] Aiba M. Kurokawa H. Onoda Y. Oguro M. Nakashizuka T. Masaki T. (2016). Context-dependent changes in the functional composition of tree communities along successional gradients after land-use change. J. Ecol. 104 (5), 1347–1356. doi: 10.1111/1365-2745.12597

[B2] Bachelot B. Alonso-Rodriguez A. M. Aldrich-Wolfe L. Cavaleri M. A. Reed S. C. Wood T. E. (2020). Altered climate leads to positive density-dependent feedbacks in a tropical wet forest. Global Change Biol. 26 (6), 3417–3428. doi: 10.1111/gcb.15087 32196863

[B3] Badano E. I. Sánchez-Montes de Oca E. J. (2022). Seed fate, seedling establishment and the role of propagule size in forest regeneration under climate change conditions. For. Ecol. Manage. 503, 119776. doi: 10.1016/j.foreco.2021.119776

[B4] Baeten L. Hermy M. Verheyen K. (2009). Environmental limitation contributes to the differential colonization capacity of two forest herbs. J. Vegetat. Sci. 20 (2), 209–223. doi: 10.1111/j.1654-1103.2009.05595.x

[B5] Bagchi R. Gallery R. E. Gripenberg S. Gurr S. J. Narayan L. Addis C. E.. (2014). Pathogens and insect herbivores drive rainforest plant diversity and composition. Nature 506 (7486), 85–88. doi: 10.1038/nature12911 24463522

[B6] Baldocchi D. D. Ma S. Rambal S. Misson L. Ourcival J. M. Limousin J. M.. (2010). On the differential advantages of evergreenness and deciduousness in mediterranean oak woodlands: a flux perspective. Ecol. Appl. 20 (6), 1583–1597. doi: 10.1890/08-2047.1 20945761

[B7] Barbosa E. R. van Langevelde F. Tomlinson K. W. Carvalheiro L. G. Kirkman K. de Bie S.. (2014). Tree species from different functional groups respond differently to environmental changes during establishment. Oecologia 174 (4), 1345–1357. doi: 10.1007/s00442-013-2853-y 24337711

[B8] Benitez-Malvido J. (1998). Impact of forest fragmentation on seedling abundance in a tropical rain forest. Conserv. Biol. 12 (2), 380–389. doi: 10.1046/j.1523-1739.1998.96295.x

[B9] Benitez-Malvido J. Martinez-Ramos M. (2003). Impact of forest fragmentation on understory plant species richness in Amazonia. Conserv. Biol. 17 (2), 389–400. doi: 10.1046/j.1523-1739.2003.01120.x

[B10] Bertness M. D. Callaway R. (1994). Positive interactions in communities. Trends Ecol. Evol. 9 (5), 191–193. doi: 10.1016/0169-5347(94)90088-4 21236818

[B11] Bond-Lamberty B. Rocha A. V. Calvin K. Holmes B. Wang C. Goulden M. L. (2014). Disturbance legacies and climate jointly drive tree growth and mortality in an intensively studied boreal forest. Global Change Biol. 20 (1), 216–227. doi: 10.1111/gcb.12404 24115380

[B12] Bruna E. M. (1999). Seed germination in rainforest fragments. Nature 402 (6758), 139–139. doi: 10.1038/45963 10647004

[B13] Bykova O. Chuine I. Morin X. Higgins S. I. (2012). Temperature dependence of the reproduction niche and its relevance for plant species distributions. J. Biogeog. 39 (12), 2191–2200. doi: 10.1111/j.1365-2699.2012.02764.x

[B14] Camargo J. L. C. Kapos V. (1995). Complex edge effects on soil moisture and microclimate in central Amazonian forest. J. Trop. Ecol. 11 (2), 205–221. doi: 10.1017/S026646740000866X

[B15] Chesson P. (2000). Mechanisms of maintenance of species diversity. Annu. Rev. Ecol. Systemat. 31 (1), 343–366. doi: 10.1146/annurev.ecolsys.31.1.343

[B16] Chidumayo E. N. (2008). Implications of climate warming on seedling emergence and mortality of African savanna woody plants. Plant Ecol. 198 (1), 61–71. doi: 10.1007/s11258-007-9385-7

[B17] Clark J. S. Iverson L. Woodall C. W. Allen C. D. Bell D. M. Bragg D. C.. (2016). The impacts of increasing drought on forest dynamics, structure, and biodiversity in the united states. Global Change Biol. 22 (7), 2329–2352. doi: 10.1111/gcb.13160 26898361

[B18] Coley P. D. Bryant J. P. Chapin F. S. 3rd (1985). Resource availability and plant antiherbivore defense. Science 230 (4728), 895–899. doi: 10.1126/science.230.4728.895 17739203

[B19] Comita L. S. Condit R. Hubbell S. P. (2007). Developmental changes in habitat associations of tropical trees. J. Ecol. 95 (3), 482–492. doi: 10.1111/j.1365-2745.2007.01229.x

[B20] Connell J. (1971). On the role of the natural enemies in preventing competitive exclusion in some marine animals and in rain forest trees. Dynam. populat., 298–312.

[B21] Connell J. H. Tracey J. Webb L. J. (1984). Compensatory recruitment, growth, and mortality as factors maintaining rain forest tree diversity. Ecol. Monogr. 54 (2), 141–164. doi: 10.2307/1942659

[B22] Davies K. F. Margules C. R. Lawrence J. F. (2004). A synergistic effect puts rare, specialized species at greater risk of extinction. Ecology 85 (1), 265–271. doi: 10.1890/03-0110

[B23] Debinski D. M. Holt R. D. (2000). A survey and overview of habitat fragmentation experiments. Conserv. Biol. 14 (2), 342–355. doi: 10.1046/j.1523-1739.2000.98081.x

[B24] De Chazal J. Rounsevell M. D. A. (2009). Land-use and climate change within assessments of biodiversity change: a review[J]. Glob. Environ. Chang. 19 (2), 306–315. doi: 10.1016/j.gloenvcha.2008.09.007

[B25] Denslow J. S. (1980). Patterns of plant species diversity during succession under different disturbance regimes. Oecologia 46 (1), 18–21. doi: 10.1007/BF00346960 28310620

[B26] Dirzo R. Young H. S. Galetti M. Ceballos G. Isaac N. J. Collen B. (2014). Defaunation in the anthropocene. Science 345 (6195), 401–406. doi: 10.1126/science.1251817 25061202

[B27] Diversity, S.o.t.C.o.B (2020). "Global biodiversity outlook 5" (Montreal, Canada: The UN Convention on Biological Diversity (CBD)).

[B28] Ebeling A. Strauss A. T. Adler P. B. Arnillas C. A. Isabel C. Biederman L. A.. (2021). Nutrient enrichment increases invertebrate herbivory and pathogen damage in grasslands. J. Ecol. 110 (2), 327–339. doi: 10.1111/1365-2745.13801

[B29] Ewers R. M. Didham R. K. (2006). Confounding factors in the detection of species responses to habitat fragmentation. Biol. Rev. 81 (1), 117–142. doi: 10.1017/S1464793105006949 16318651

[B30] Fahrig L. (2003). Effects of habitat fragmentation on biodiversity. Annu. Rev. ecol. evol. systemat. 34 (1), 487–515. doi: 10.1146/annurev.ecolsys.34.011802.132419

[B31] Fang X. F. Shen G. C. Yang Q. S. Liu H. M. Ma Z. P. Deane D. C.. (2017). Habitat heterogeneity explains mosaics of evergreen and deciduous trees at local-scales in a subtropical evergreen broad-leaved forest. J. Vegetat. Sci. 28 (2), 379–388. doi: 10.1111/jvs.12496

[B32] Givnish T. J. (2002). Adaptive significance of evergreen vs. deciduous leaves: solving the triple paradox. Silva Fennica 36 (3), 703–743. doi: 10.14214/sf.535

[B33] González-Di Pierro A. M. Benítez-Malvido J. Méndez-Toribio M. Zermeño I. Arroyo-Rodríguez V. Stoner K. E.. (2011). Effects of the physical environment and primate gut passage on the early establishment of ampelocera hottlei standley in rain forest fragments. Biotropica 43 (4), 459–466. doi: 10.1111/j.1744-7429.2010.00734.x

[B34] Greenlee J. T. Callaway R. M. (1996). Abiotic stress and the relative importance of interference and facilitation in montane bunchgrass communities in western montana. Am. Nat. 148 (2), 386–396. doi: 10.1086/285931

[B35] Grubb P. J. (1977). Maintenance of species-richness in plant communities - importance of regeneration niche. Biol. Rev. 52 (1), 107–145. doi: 10.1111/j.1469-185X.1977.tb01347.x

[B36] Harper J. L. (1977). Population biology of plants (London: Academic Press).

[B37] Harrison R. D. Tan S. Plotkin J. B. Slik F. Detto M. Brenes T.. (2013). Consequences of defaunation for a tropical tree community. Ecol. Lett. 16 (5), 687–694. doi: 10.1111/ele.12102 23489437

[B38] Hart S. P. Usinowicz J. Levine J. M. (2017). The spatial scales of species coexistence. Nat. Ecol. Evol. 1 (8), 1066–1073. doi: 10.1038/s41559-017-0230-7 29046584

[B39] Hillyer R. Silman M. R. (2010). Changes in species interactions across a 2.5 km elevation gradient: effects on plant migration in response to climate change. Global Change Biol. 16 (12), 3205–3214. doi: 10.1111/j.1365-2486.2010.02268.x

[B40] Hu G. Feeley K. J. Wu J. G. Xu G. F. Yu M. J. (2011). Determinants of plant species richness and patterns of nestedness in fragmented landscapes: evidence from land-bridge islands. Landscape Ecol. 26 (10), 1405–1417. doi: 10.1007/s10980-011-9662-7

[B41] Hu G. Wilson M. Zhou B. B. Shang C. Yu M. Wu J. (2021). Spatiotemporal patterns and ecological consequences of a fragmented landscape created by damming. PeerJ 9, e11416. doi: 10.7717/peerj.11416 34055485 PMC8142928

[B42] Hutchinson G. E. (1961). The paradox of the plankton. Am. Nat. 95 (882), 137–145. doi: 10.1086/282171

[B43] Ibanez I. Katz D. S. W. Lee B. R. (2017). The contrasting effects of short-term climate change on the early recruitment of tree species. Oecologia 184 (3), 701–713. doi: 10.1007/s00442-017-3889-1 28573380

[B44] Ibanez I. Schupp E. W. (2001). Positive and negative interactions between environmental conditions affecting cercocarpus ledifolius seedling survival. Oecologia 129 (4), 543–550. doi: 10.1007/s004420100757 24577694

[B45] Inman-Narahari F. Ostertag R. Hubbell S. P. Giardina C. P. Cordell S. Sack L. (2016). Density-dependent seedling mortality varies with light availability and species abundance in wet and dry Hawaiian forests. J. Ecol. 104 (3), 773–780. doi: 10.1111/1365-2745.12553

[B46] Janzen D. H. (1970). Herbivores and the number of tree species in tropical forests. Am. Nat. 104 (940), 501–528. doi: 10.1086/282687

[B47] Jin Y. Russo S. E. Yu M. J. (2018). Effects of light and topography on regeneration and coexistence of evergreen and deciduous tree species in a Chinese subtropical forest. J. Ecol. 106 (4), 1634–1645. doi: 10.1111/1365-2745.12911

[B48] Joet T. Ourcival J. M. Dussert S. (2013). Ecological significance of seed desiccation sensitivity in quercus ilex. Ann. Of Bot. 111 (4), 693–701. doi: 10.1093/aob/mct025 23388882 PMC3605958

[B49] Kapos V. (1989). Effects of isolation on the water status of forest patches in the Brazilian Amazon. J. Trop. Ecol. 5 (2), 173–185. doi: 10.1017/S0266467400003448

[B50] Kitajima K. Cordero R. A. Wright S. J. (2013). Leaf life span spectrum of tropical woody seedlings: effects of light and ontogeny and consequences for survival. Ann. Bot. 112 (4), 685–699. doi: 10.1093/aob/mct036 23532047 PMC3736767

[B51] Kooyman R. M. Zanne A. E. Gallagher R. V. Cornwell W. Rossetto M. O'Connor P.. (2013). Effects of growth form and functional traits on response of woody plants to clearing and fragmentation of subtropical rainforest. Conserv. Biol. 27 (6), 1468–1477. doi: 10.1111/cobi.12088 23869490

[B52] Kroiss S. J. Hillerslambers J. (2015). Recruitment limitation of long-lived conifers: implications for climate change responses. Ecology 96 (5), 1286–1297. doi: 10.1890/14-0595.1 26236842

[B53] Laurance W. F. (2004). Forest-climate interactions in fragmented tropical landscapes. Philos. Trans. R. Soc. B: Biol. Sci. 359 (1443), 345–352. doi: 10.1098/rstb.2003.1430 PMC1693331 15212089

[B54] Laurance W. F. Camargo J. L. Fearnside P. M. Lovejoy T. E. Williamson G. B. Mesquita R. C.. (2018). An a mazonian rainforest and its fragments as a laboratory of global change. Biol. Rev. 93 (1), 223–247. doi: 10.1111/brv.12343 28560765

[B55] Laurance W. F. Delamonica P. Laurance S. G. Vasconcelos H. L. Lovejoy T. E. (2000). Rainforest fragmentation kills big trees. Nature 404 (6780), 836. doi: 10.1038/35009032 10786782

[B56] Laurance W. F. Nascimento H. E. Laurance S. G. Andrade A. Ribeiro J. E. Giraldo J. P.. (2006). Rapid decay of tree-community composition in Amazonian forest fragments. Proc. Natl. Acad. Sci. United States America 103 (50), 19010–19014. doi: 10.1073/pnas.0609048103 PMC1682011 17148598

[B57] Laurance W. F. Yensen E. (1991). Predicting the impacts of edge effects in fragmented habitats. Biol. Conserv. 55 (1), 77–92. doi: 10.1016/0006-3207(91)90006-U

[B58] Leck M. A. Parker V. T. Simpson R. L. E. (1989). Ecology of soil seed banks (San Diego, California, USA: Academic Press).

[B59] Liu J. Coomes D. A. Gibson L. Hu G. Liu J. Luo Y.. (2019a). Forest fragmentation in China and its effect on biodiversity. Biol. Rev. Cambridge Philos. Soc. 94 (5), 1636–1657. doi: 10.1111/brv.12519 31058438

[B60] Liu J. J. Coomes D. A. Hu G. Liu J. L. Yu J. J. Luo Y. Q.. (2019c). Larger fragments have more late-successional species of woody plants than smaller fragments after 50 years of secondary succession. J. Ecol. 107 (2), 582–594. doi: 10.1111/1365-2745.13071

[B61] Liu J. Matthews T. J. Zhong L. Liu J. Wu D. Yu M.. (2019b). Environmental filtering underpins the island species–area relationship in a subtropical anthropogenic archipelago. J. Ecol. 108 (2), 424–432. doi: 10.1111/1365-2745.13272

[B62] Liu J. L. Vellend M. Wang Z. H. Yu M. J. (2018). High beta diversity among small islands is due to environmental heterogeneity rather than ecological drift. J. Biogeog. 45 (10), 2252–2261. doi: 10.1111/jbi.13404

[B63] Macarthur R. Levins R. (1967). Limiting similarity convergence and divergence of coexisting species. Am. Nat. 101 (921), 377–385. doi: 10.1086/282505

[B64] Malcolm J. R. (1994). Edge effects in central Amazonian forest fragments. Ecology 75 (8), 2438–2445. doi: 10.2307/1940897

[B65] Mantyka-Pringle C. S. Martin T. G. Rhodes J. R. (2011). Interactions between climate and habitat loss effects on biodiversity: a systematic review and meta-analysis. Global Change Biol. 18 (4), 1239–1252. doi: 10.1111/gcb.12148

[B66] Martin P. A. Newton A. C. Bullock J. M. (2014). Carbon pools recover more quickly than plant biodiversity in tropical secondary forests (vol 281, 20140303, 2014). Proc. R. Soc. B-Biol. Sci. 281 (1782), 20140303. doi: 10.1098/rspb.2014.0303 PMC3826225 24197410

[B67] Matlack G. R. (1994). Vegetation dynamics of the forest edge–trends in space and successional time. J. Ecol. 82, 113–123. doi: 10.2307/2261391

[B68] McKone M. J. Kelly D. Lee W. G. (1998). Effect of climate change on mast-seeding species: frequency of mass flowering and escape from specialist insect seed predators. Global Change Biol. 4 (6), 591–596. doi: 10.1046/j.1365-2486.1998.00172.x

[B69] Milici V. R. Dalui D. Mickley J. G. Bagchi R. (2020). Responses of plant-pathogen interactions to precipitation: implications for tropical tree richness in a changing world. J. Ecol. 108 (5), 1800–1809. doi: 10.1111/1365-2745.13373

[B70] Moles A. T. Westoby M. (2004). Seedling survival and seed size: a synthesis of the literature. J. Ecol. 92 (3), 372–383. doi: 10.1111/j.0022-0477.2004.00884.x

[B71] Opdam P. Wascher D. (2004). Climate change meets habitat fragmentation: linking landscape and biogeographical scale levels in research and conservation. Biol. Conserv. 117 (3), 285–297. doi: 10.1016/j.biocon.2003.12.008

[B72] Peña-Domene M. Howe H. F. Cruz-León E. Jiménez-Rolland R. Lozano-Huerta C. Martínez-Garza C. (2016). Seed to seedling transitions in successional habitats across a tropical landscape. Oikos 126 (3), 410–419. doi: 10.1111/oik.03394

[B73] Perez-Ramos I. M. Volaire F. Fattet M. Blanchard A. Roumet C. (2013). Tradeoffs between functional strategies for resource-use and drought-survival in Mediterranean rangeland species. Environ. Exp. Bot. 87, 126–136. doi: 10.1016/j.envexpbot.2012.09.004

[B74] Perez-Ruiz C. L. Badano E. I. Rodas-Ortiz J. P. Delgado-Sanchez P. Flores J. Douterlungne D.. (2018). Climate change in forest ecosystems: a field experiment addressing the effects of raising temperature and reduced rainfall on early life cycle stages of oaks. Acta Oecologica-Internat. J. Ecol. 92, 35–43. doi: 10.1016/j.actao.2018.08.006

[B75] Pitelka L. F., and the Plant Migration Workshop Group (1997). Plant migration and climate change: a more realistic portrait of plant migration is essential to predicting biological responses to global warming in a world drastically altered by human activity. Am. Scientist 85 (5), 464–473.

[B76] Porensky L. M. (2011). When edges meet: interacting edge effects in an African savanna. J. Ecol. 99 (4), 923–934. doi: 10.1111/j.1365-2745.2011.01824.x

[B77] Renton M. Shackelford N. Standish R. J. (2013). How will climate variability interact with long-term climate change to affect the persistence of plant species in fragmented landscapes? Environ. Conserv. 41 (2), 110–121. doi: 10.1017/s0376892913000490

[B78] Rosenzweig M. L. Rosenzweig M. L. (1995). Species diversity in space and time (New York, USA: Cambridge University Press).

[B79] Rother D. C. Jordano P. Rodrigues R. R. Pizo M. A. (2013). Demographic bottlenecks in tropical plant regeneration: a comparative analysis of causal influences. Perspect. Plant Ecol. Evol. Systemat. 15 (2), 86–96. doi: 10.1016/j.ppees.2012.12.004

[B80] Sarmiento F. O. (1997). Arrested succession in pastures hinders regeneration of tropandean forests and shreds mountain landscapes. Environ. Conserv. 24 (1), 14–23. doi: 10.1017/S0376892997000052

[B81] Saunders D. A. Hobbs R. J. Margules C. R. (1991). Biological consequences of ecosystem fragmentation - a review. Conserv. Biol. 5 (1), 18–32. doi: 10.1111/j.1523-1739.1991.tb00384.x

[B82] Sizer N. C. (1992). The impact of edge formation on regeneration and litterall in a tropical rain forest fragment in Amazonia (Cambridge, England: University of Cambridge).

[B83] Slik J. W. Bernard C. S. Breman F. C. VAN Beek. M. Salim A. Sheil D. (2008). Wood density as a conservation tool: quantification of disturbance and identification of conservation-priority areas in tropical forests. Conserv. Biol. 22 (5), 1299–1308. doi: 10.1111/j.1523-1739.2008.00986.x 18637916

[B84] Sousa T. R. Costa F. R. C. Bentos T. V. Leal N. Mesquita R. C. G. Ribeiro I. O. (2017). The effect of forest fragmentation on the soil seed bank of central Amazonia. For. Ecol. Manage. 393, 105–112. doi: 10.1016/j.foreco.2017.03.020

[B85] Swaine M. D. (1996). The ecology of tropical forest tree seedlings (New York, USA: Parthenon Publishing Group Ltd).

[B86] Tabarelli M. Aguiar A. V. Girao L. C. Peres C. A. Lopes A. V. (2010). Effects of pioneer tree species hyperabundance on forest fragments in northeastern Brazil. Conserv. Biol. 24 (6), 1654–1663. doi: 10.1111/j.1523-1739.2010.01529.x 20497203

[B87] Tabarelli M. Pinto L. P. Silva J. M. C. Hirota M. Bede L. (2005). Challenges and opportunities for biodiversity conservation in the Brazilian Atlantic forest. Conserv. Biol. 19 (3), 695–700. doi: 10.1111/j.1523-1739.2005.00694.x

[B88] Tang C. Q. Ohsawa M. (2002). Coexistence mechanisms of evergreen, deciduous and coniferous trees in a mid-montane mixed forest on mt. emei, sichuan, China. Plant Ecol. 161 (2), 215–230. doi: 10.1023/A:1020395830795

[B89] Tian Y. Jin Y. Wang Z. SU X. Hu G. Xu L.. (2016). Seedling dynamics of shade tolerant and intolerant woody plants in the Masson pine forests on islands of the Thousand Island Lake. J. ZheJIang Univ. (Sci. Ed.) 43 (4), 426–435. doi: 10.3785/j.issn.1008-9497.2016.04.009

[B90] Tielborger K. Kadmon R. (2000). Temporal environmental variation tips the balance between facilitation and interference in desert plants. Ecology 81 (6), 1544–1553. doi: 10.1890/0012-9658(2000)081[1544:Tevttb]2.0.Co;2

[B91] Turner M. G. Baker W. L. Peterson C. J. Peet R. K. (1998). Factors influencing succession: lessons from large, infrequent natural disturbances. Ecosystems 1 (6), 511–523. doi: 10.1007/s100219900047

[B92] Wang X. H. Kent M. Fang X. F. (2007). Evergreen broad-leaved forest in eastern China: its ecology and conservation and the importance of resprouting in forest restoration. For. Ecol. Manage. 245 (1-3), 76–87. doi: 10.1016/j.foreco.2007.03.043

[B93] Way D. A. Oren R. (2010). Differential responses to changes in growth temperature between trees from different functional groups and biomes: a review and synthesis of data. Tree Physiol. 30 (6), 669–688. doi: 10.1093/treephys/tpq015 20368338

[B94] Wilcox B. A. Murphy D. D. (1985). Conservation strategy: the effects of fragmentation on extinction. Am. Nat. 125 (6), 879–887. doi: 10.1086/284386

[B95] Yu M. J. Hu G. Feeley K. J. Wu J. G. Ding P. (2012). Richness and composition of plants and birds on land-bridge islands: effects of island attributes and differential responses of species groups. J. Biogeog. 39 (6), 1124–1133. doi: 10.1111/j.1365-2699.2011.02676.x

