# Supplementary material for: Climate and fragment area jointly affect the annual dynamics of seedlings in different functional groups in the Thousand Island Lake
Source: Front Plant Sci. 2023 Jun 14;14:1200520. doi: 10.3389/fpls.2023.1200520 (PMC10303124; doi:10.3389/fpls.2023.1200520)
Supplement: Supplementary file 1 [file DataSheet_1.docx]

Supplementary Material

# Supplementary Figures and Tables

## Supplementary Tables

**Table S1.** Island parameter and plot setting of the 29 study islands in the Thousand Island Lake, China.

| **Island** | **Island Type** | **Area (ha)** | **Number of Seed Traps** | **Number of Seedling Plots** |
| --- | --- | --- | --- | --- |
| 34 | s | 0.0788 | 3 | 6 |
| 68 | s | 0.0996 | 3 | 3 |
| 59 | s | 0.1821 | 3 | 6 |
| 36 | s | 0.1888 | 3 | 9 |
| 60 | s | 0.259 | 3 | 6 |
| 50 | s | 0.289 | 3 | 12 |
| 32 | s | 0.337 | 3 | 9 |
| 33 | s | 0.4027 | 3 | 6 |
| 73 | s | 0.434 | 3 | 9 |
| 69 | s | 0.476 | 3 | 14 |
| 14 | s | 0.488 | 3 | 9 |
| 35 | s | 0.5279 | 6 | 12 |
| 15 | s | 0.5884 | 6 | 12 |
| 74 | s | 0.6228 | 3 | 6 |
| 72 | s | 0.6325 | 6 | 12 |
| 58 | s | 0.7149 | 6 | 18 |
| 78 | s | 0.9225 | 6 | 15 |
| 31 | s | 0.927 | 6 | 14 |
| 113 | m | 1.156 | 6 | 18 |
| 37 | m | 1.3589 | 12 | 27 |
| 75 | m | 1.4217 | 6 | 9 |
| 64 | m | 1.5602 | 12 | 21 |
| 63 | m | 1.819 | 12 | 24 |
| 77 | m | 3.0339 | 12 | 21 |
| 43 | m | 4.0584 | 12 | 33 |
| 117 | m | 9.7287 | 24 | 39 |
| B7 | l | 29.0535 | 24 | 30 |
| B6 | l | 51.8885 | 24 | 36 |
| JSD | l | 1158.1 | 14 | 33 |
| JSN | l | 1158.1 | 10 | 30 |
| Total |  |  | 240 | 499 |

**Table S2.** List of the plant species recorded in seedling plots and seed traps on 29 study islands in the Thousand Island Lake, China. Species are assigned into functional groups with two categories of leave life form and shade tolerance.

| Species | Tree abundance | Leaf life form | Shade tolerance |
| --- | --- | --- | --- |
| *Loropetalum chinense* | 49388 | evergreen | tolerant |
| *Vaccinium carlesii* | 22880 | evergreen | tolerant |
| *Pinus massoniana* | 11468 | evergreen | intolerant |
| *Rhododendron simsii* | 8416 | deciduous | intolerant |
| *Eurya muricata* | 6421 | evergreen | tolerant |
| *Dalbergia hupeana* | 3537 | deciduous | tolerant |
| *Quercus serrata* | 2406 | deciduous | intolerant |
| *Lyonia ovalifolia var. hebecarpa* | 2039 | deciduous | intolerant |
| *Juniperus formosana* | 1961 | evergreen | intolerant |
| *Schima superba* | 1554 | evergreen | tolerant |
| *Camellia fraterna* | 1407 | evergreen | tolerant |
| *Quercus fabrei* | 1340 | deciduous | intolerant |
| *Rhaphiolepis indica* | 1151 | evergreen | tolerant |
| *Cunninghamia lanceolata* | 1058 | evergreen | intolerant |
| *Lindera aggregata* | 903 | evergreen | tolerant |
| *Ilex chinensis* | 830 | evergreen | tolerant |
| *Lithocarpus glaber* | 797 | evergreen | tolerant |
| *Castanopsis sclerophylla* | 643 | evergreen | tolerant |
| *Symplocos paniculata* | 534 | deciduous | intolerant |
| *Quercus acutissima* | 486 | deciduous | intolerant |
| *Symplocos sumuntia* | 405 | evergreen | tolerant |
| *Liquidambar formosana* | 398 | deciduous | intolerant |
| *Styrax faberi* | 331 | deciduous | tolerant |
| *Diospyros kaki* | 320 | deciduous | tolerant |
| *Albizia kalkora* | 200 | deciduous | intolerant |
| *Gardenia jasminoides* | 196 | evergreen | tolerant |
| *Symplocos stellaris* | 196 | evergreen | tolerant |
| *Vitex negundo var. cannabifolia* | 191 | deciduous | intolerant |
| *Lindera glauca* | 153 | deciduous | intolerant |
| *Ilex rotunda* | 133 | evergreen | tolerant |
| *Styrax dasyanthus* | 108 | deciduous | tolerant |
| *Frangula crenata* | 97 | deciduous | intolerant |
| *Premna microphylla* | 90 | deciduous | tolerant |
| *Syzygium buxifolium* | 89 | evergreen | tolerant |
| *Triadica sebifera* | 24 | deciduous | tolerant |
| *Alangium kurzii* | 19 | deciduous | tolerant |
| *Broussonetia kazinoki* | 18 | deciduous | intolerant |
| *Glochidion puberum* | 12 | deciduous | tolerant |
| *Rhus chinensis* | 10 | deciduous | intolerant |
| *Ilex cornuta* | 10 | evergreen | tolerant |
| *Serissa japonica* | 3 | deciduous | intolerant |
| *Xylosma racemosum* | 3 | deciduous | tolerant |
| *Alangium chinense* | 0 | deciduous | tolerant |
| *Callicarpa giraldii* | 0 | deciduous | intolerant |
| *Elaeagnus pungens* | 0 | evergreen | tolerant |
| *Indigofera tinctoria* | 0 | deciduous | intolerant |
| *Lespedeza bicolor* | 0 | deciduous | intolerant |
| *Rosa laevigata* | 0 | evergreen | intolerant |
| *Sageretia thea* | 0 | evergreen | tolerant |
| *Trema cannabina var. dielsiana* | 0 | deciduous | intolerant |

## Supplementary Figures


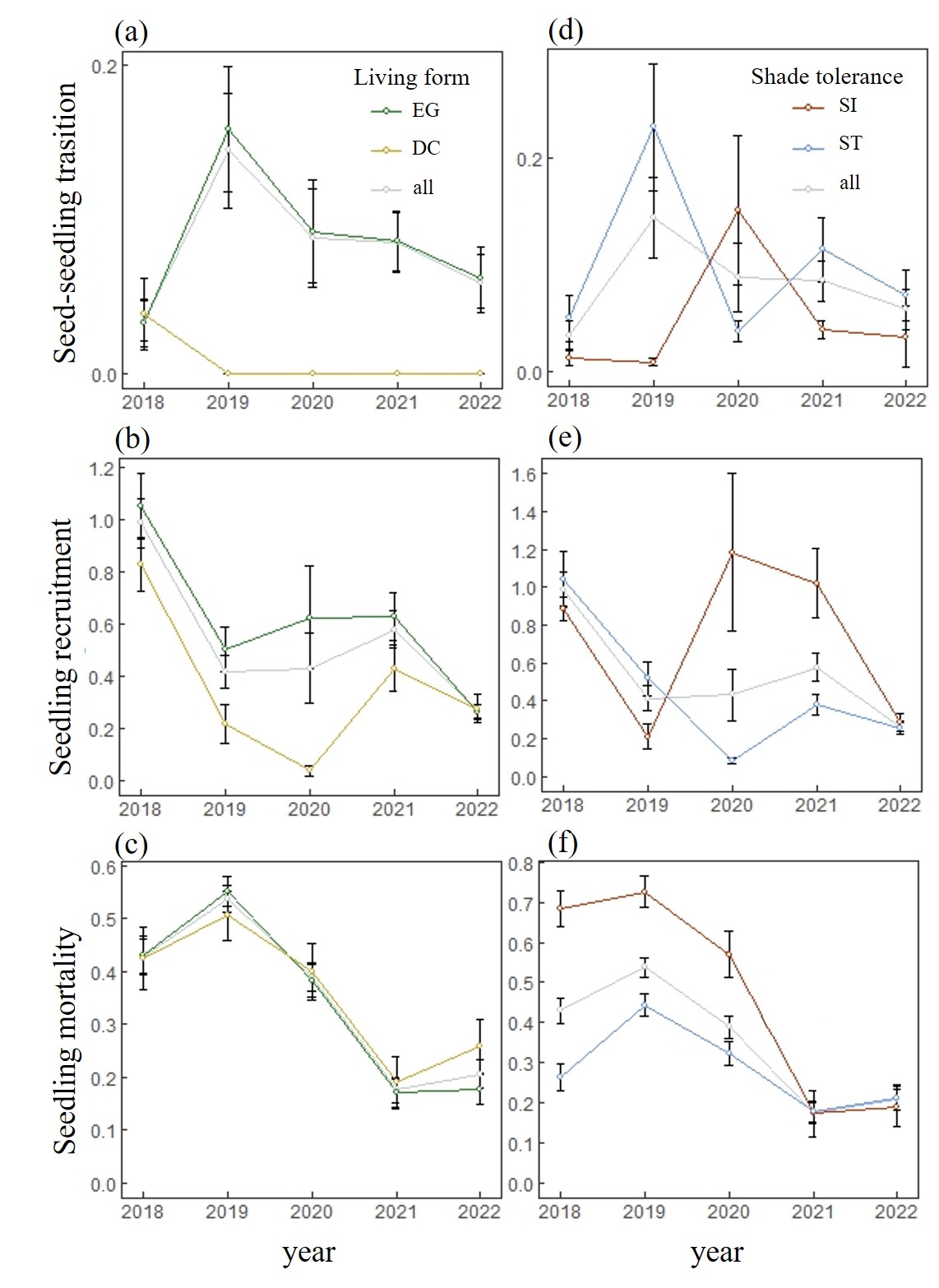


**Figure S1.** Seedling dynamics without excluding gymnosperms species (seed-seedling transition, seedling recruitment and seedling mortality) from 2018 to 2022.


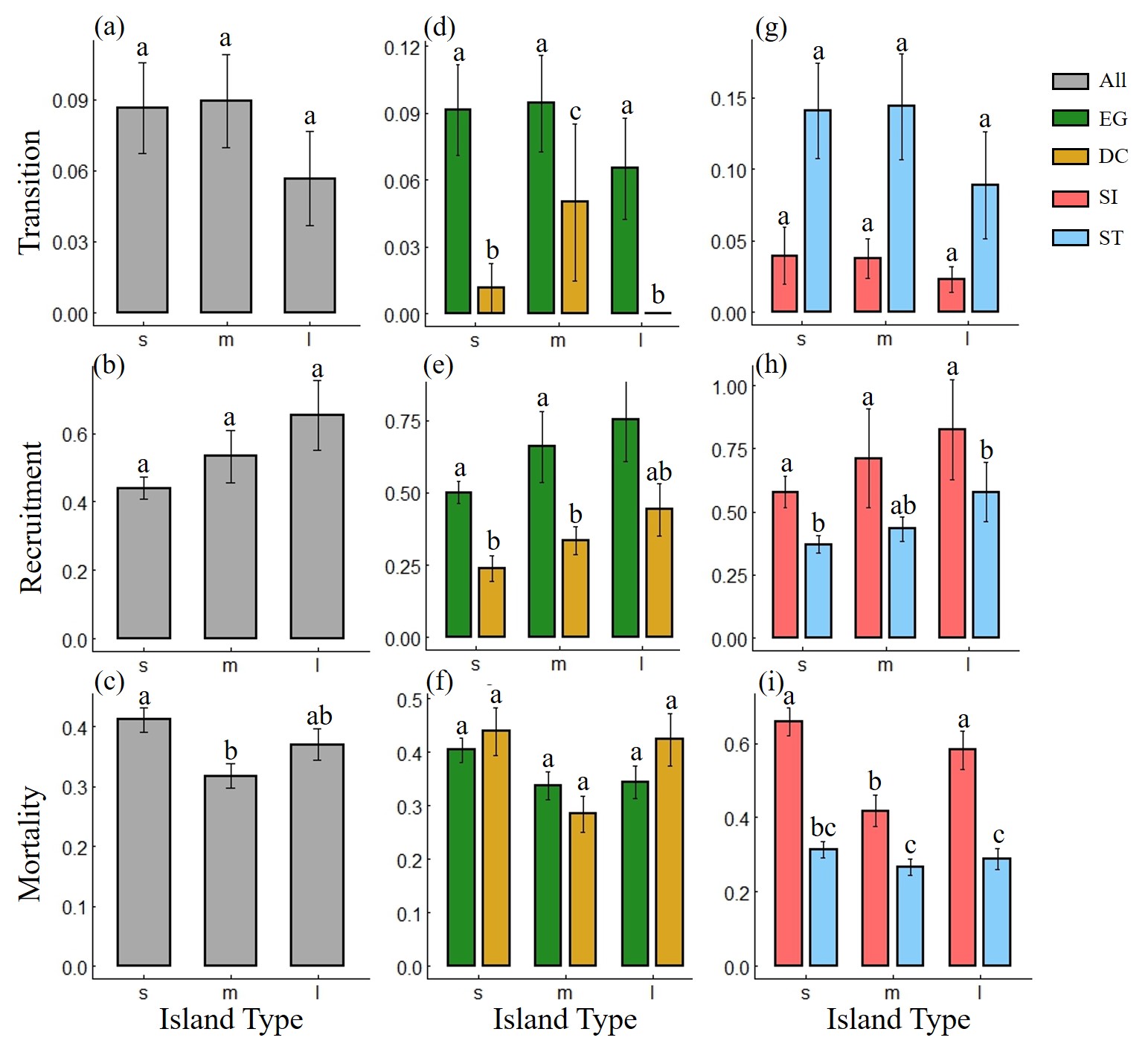


**Figure S2.** Seedling dynamics without excluding gymnosperms species (seed-seedling transition, a, d, g; seedling recruitment, b, e, h; and seedling mortality, c, f, i) of different functional group from 2018 to 2022. Seedling dynamic of all species (grey), EG (green) and deciduous (yellow) species, Shade-intolerance (red) and shade-tolerance (blue) species were shown. Different letters between functional groups or island types indicated significant difference.


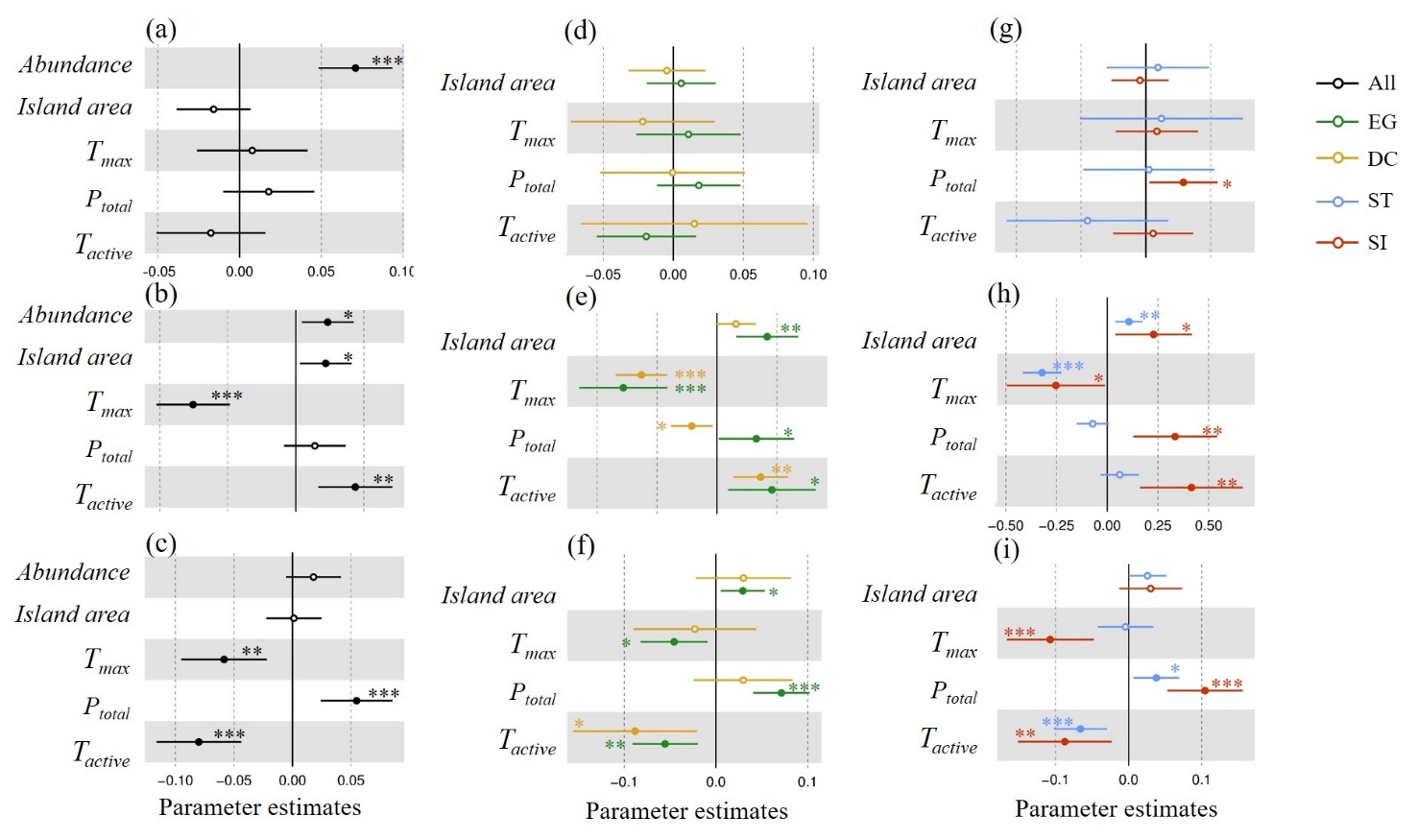


**Figure S3.** The relative influence of potential factors (the community abundance (Abundance), island area, annual maximum temperature (*T_max_*), annual precipitation (*P_total_*) and active accumulated temperature (*T_active_*)) on the transition (a, d, g), recruitment (b, e, h) and mortality (c, f, i) rate of seedlings of evergreen (EG, green), deciduous (DC, yellow), shade-intolerat (SI, red), shade-tolerant (ST, blue), and all (black) species without excluding gymnosperms species. Solid points with asterisk marks indicate a significant effect (significant codes for p-value: p= 0~ 0.001 “***”, p= 0.001~ 0.01 “**”, p= 0.01~ 0.05 “*”). If the coefficient value of the explanatory variable is <0, it means a significant negative correlation. The parameter estimate <0 indicated a significant negative effect. The parameter estimate intersects with 0 indicated an insignificant effect. The parameter estimate >0 indicates a significant positive effect.


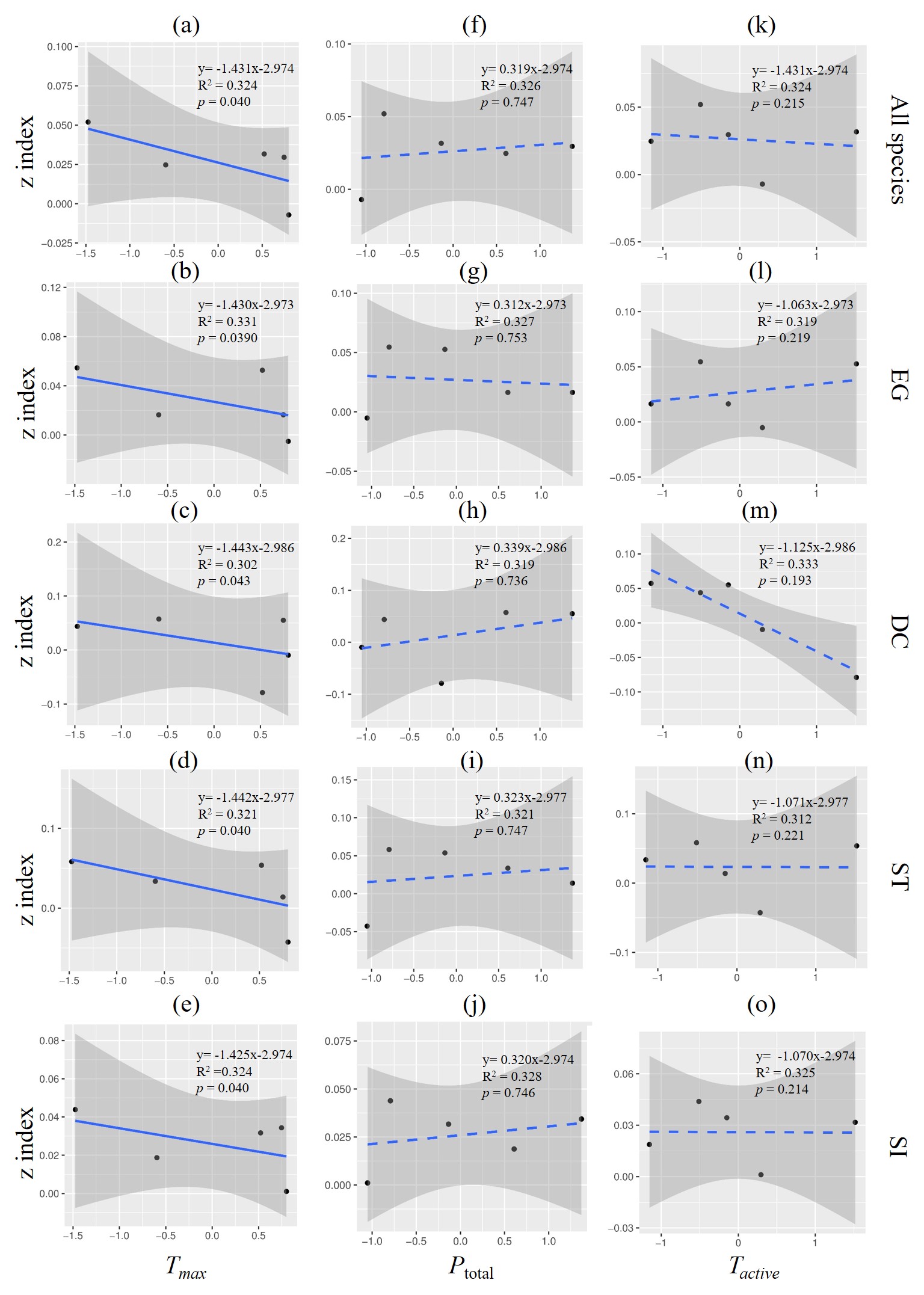


**Figure S4.** Linear regression correlation between *z*-value and annual maximum temperature (*T_max_*: a, b, c, d, e), annual precipitation (*P_total_*, f, g, h, i, j), and the annual active accumulated temperature *(T_active_*: k, l, m, n, o) for all species (a, f, k), evergreen species (b, g, l), deciduous species (c, h, m), shade-tolerant species (d, i, n), and shade-intolerant species (e, j, o) without excluding gymnosperms species. To be noted, the *p*-value in this figure was calculated based on the results of LM with time offset.
